# Blocking and Deblocking of Diisocyanate to Synthesize Polyurethanes

**DOI:** 10.3390/polym13172875

**Published:** 2021-08-27

**Authors:** Sourita Jana, Debasis Samanta, Mir Muhammad Fahad, Sellamuthu N. Jaisankar, Hongdoo Kim

**Affiliations:** 1 Polymer Science & Technology Division, CSIR-Central Leather Research Institute, Adyar, Chennai 600020, India; janasour@gmail.com (S.J.); debasis.samanta@gmail.com (D.S.); 2 University of Madras, Chennai 600005, India; 3 Department of Advanced Materials Engineering for Information and Electronics, College of Engineering, Kyung Hee University, Yongin-si 17104, Korea; fahad@khu.ac.kr

**Keywords:** toluene diisocyanate, 3-(4-bromophenyl)-1H-pyrazole, blocking agent, blocked adduct, deblocks, polyol, polyurethane, coatings

## Abstract

Diisocyanates, particularly toluene diisocyanate (TDI), are useful for the preparation of various polyurethanes with specific applications as leather-like materials, adhesives and insoles, etc. Blocking agents can be used for the operational simplicity and to reduce the hazards of TDI. In this paper, we reported the use of 3-(4-bromo-phenyl)-1H-pyrazole to block toluene diisocyanate (TDI). FTIR, NMR, thermogravimetric analysis, contact angle analysis and differential scanning calorimetry (DSC) were used for the characterization. The effectiveness of the blocking was confirmed by spectroscopic techniques. The DSC thermogram showed that blocked adducts deblock at 240 °C, causing the regeneration of TDI, and causing the diisocyanates to react with polyols of different molecular weights, forming polyurethanes. The characterization of the polyurethanes was performed by infrared spectroscopy, nuclear magnetic resonance spectroscopy, thermogravimetric analysis, differential scanning calorimetry and a contact angle study.

## 1. Introduction

Polyurethanes are widely used for the manufacturing of jackets, shoe components and parts of furniture. Their flexibility, low glass transition temperature and high mechanical strength make them an attractive choice for the preparation of leather-like materials. In many cases, polyurethanes have hard segments as well as soft segments. In many cases, the soft segments which are generated from polyethylene glycol or polypropylene glycol can affect the glass transition temperatures, melting, or hydrophobicity. The choice of reagents, the process of preparations, or the surface morphologies are essential for the development of polyurethanes with better properties.

Polyurethanes are usually prepared by the step-growth polymerization of diisocianates and diols or polyols [1]. Because some of the diisocyanates are hazardous, they can be blocked with compounds with active hydrogen, so that they can be unblocked in the presence of diols to produce polyurethanes [2]. Very recently, different compounds have been used as blocking agents, such as pyrazoles and indoles, etc. [3]. Recently, 2-formyloxyethyl methacrylate, a liquid blocking agent, was used to block different isocyanates [4]. Likewise, 2,4-Toluene diisocyanate has been blocked with imidazole, 2-methylimidazole and 2-Phenyl-imidazole [5]. In this case, the addition of isocyanates with active hydrogen compounds like alcohols, amines and thiols, etc., leads to the formation of urethane, urea, thiourethane and amides. Among those, thiols react with isocyanates at a slower rate than alcohols and amines [6]. Phenol-, 2-naphthol- and 1-nitroso-2-naphthol were used as blocking agents to block toluene diisocyanate (TDI) and isophorone diisocyanate (IPDI). The 2-naphthol-blocked diisocyanate adducts were thermally less stable than the phenol-blocked diisocyanates adducts. The introduction of the nitroso group in the blocking agent reduces the dissociation temperature. ε-caprolactam has been widely used as an effective blocking agent for isocyanates [7]. Polyisocyanates blocked with caprolactam represent a commercially important class of polyols. The dissociation temperature of a polyisocyanate blocked with ε-caprolactam ranges between 130 °C and 160 °C [8]. Further, malonate and oximes, etc., have been reported as blocking agents for isocyanates [9,10]. Sodium bisulfite-blocked isophorone diisocyanate is water soluble and stable for more than 12 months at room temperature, both in a liquid and solid state [11]. The blocking of polymethylene polyphenyl isocyanates (PAPI) had been acheived by using sodium bisulfate [12]. Among the different blocking agents, pyrazole-based blocking agents have attracted special attention because of their effectiveness in blocking as well as deblocking [13]. The deblocking reactions of pyrazole-blocked isocyanate proceed through a five-center complex formation [12,14]. In this context, a group of water-dispersible blocked polyisocyanates were synthesized from toluene 2,4-diisocyanate (TDI) and isophorone diisocyanate (IPDI) using ethyl cellosolve (EC), epsilon-caprolactam (CL) and methyl ethyl ketoxime (MEKO) as blocking agents in the presence of dimethylol propionic acid [15]. The aqueous dispersions of the adducts offer good storage stability. The initial deblocking temperatures of the adducts are about 55–85 °C. Furthermore, methyl anacardate, secondary butyl anacardate and cardanol-N-hydroxyphthalimide (NHPH) were separately used as blocking agents to block 2,4-toluene diisocyanate [16]. Aqueous polyurethane dispersions have been prepared from castor oil [17], and a polyurethane coating with better adhesion and anticorrosion protection, synthesized from blocked isocyanate, has been reported recently [18]. Along the line, toluene diisocyante (TDI), isophoronediisocyanate (IPDI), hexamethylenediisocyanate (HDI) and 4,4’-diphenylmethanediisocyanate (MDI) blocked with 2-butoxyethanol have been reported separately [19].

The efficacy of the formation of blocked isocyanate depends on the structures of the isocyanate and blocking agent, the thermal stability of the isocyanate-blocking agent bond, and catalysis [20]. Along the same line, isocyanates were blocked with methyl ethyl ketoxime (MEKO) and bisulfite (BS) for the fabrication of DNA microarrays [20]. Isocyanates like hexamethyelenediisocyanate, diphenylmethane diisocyanate and isophorone diisocyanate can also be blocked with diethylene glycol monobutyl ether [21]. Imidazole-blocked 2,4-toluene diisocyanate with polyethylene glycol has been synthesized and characterized with DSC, TGA, DMA and glass transition temperature [22]. Furthermore, alkyl-pyrazole has been studied as a blocking agent [23], and polyurethanes have been synthesized from 4-bromo-1H-pyrazole-blocked HMDI [13]; polyurethane composites with nanomaterials have been reported by our group recently [24,25,26,27].

Although blocked isocyanates are useful for their non-hazardous methods, the stability and environment-friendliness of polyurethane elastomers depends on the use of aromatic or aliphatic isocyanates and chain extenders like diols, triols and anddiamines [28]. Their recyclability and properties have been studied in a few cases [29]. Furthermore, the degradation of polyurethane elastomers can happen by photo-thermal, ozonolytic, hydrolytic, chemical, enzymatic, in-vivo/in-vitro oxidative, biological, and mechanical degradation [30]. Recently we reported that biodegradable polyurethane foam is used in footware to reduce waste [31]. In this context, thermogravimetric analysis (TGA) can be conveniently used for the study of thermal stability, such as the degradation temperatures and the stages of degradations of polyurethanes. On the other hand, differential scanning calorimetry (DSC) is usually used for the study of the glass transition temperatures of polyurethane in order to understand its flexibility or application potential at different temperatures. Along the same lines, the hydrophobicity of polyurethane films can be studied by contact angle meters using water droplets. In this case, droplets on more hydrophilic polyurethane will spread, resulting in the lowering of the contact angle, while more hydrophobic polyurethane can provide a higher contact angle, often resulting in the rolling out of the water droplet from the surface. Although chemical compositions are the main factors influencing hydrophobicity, high surface roughness can cause superior hydrophobicity.

In this paper, we reported an efficient method of blocking diisocyanates to prepare polyurethanes using biocompatible polyethylene glycol. Deblocking and polyurethane formations were performed in a simple operation without using any organic solvent. Furthermore, the thus-formed polyurethanes were studied using standard methods such as TGA, DSC and a contact angle meter (vide infra) for their practical usability. Finally, the polyurethanes were successfully used as coating agents to improve hydrophobicity.

## 2. Materials and Methods

Toluene diisocyanate, polyethylene glycol-400 (PEG-400), PPG-1000, PPG-2000 and PPG-3000 were purchased from Sigma-Aldrich, Bangalore, India; 4-(3-bromophenyl)-1H-pyrazole was purchased from TCI, Japan and dibutyltindilaurate (DBTDL) was purchased from FLUKA SG, India and used as received. Solvents like chloroform and hexane are purchased from Emparta ER and used after purification.

**Scheme 1:**
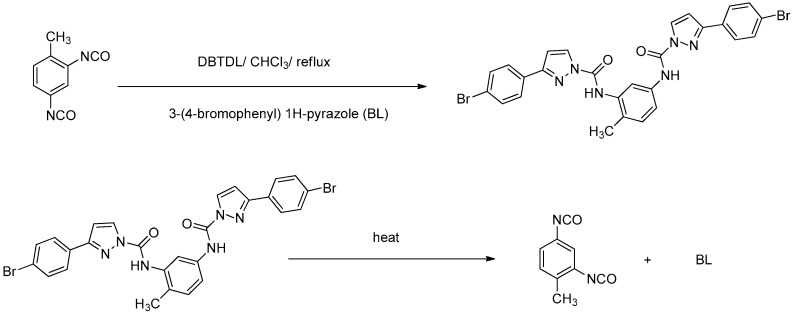
Blocking reactions of isocyanates with 3-(4-bromophenyl)-1H-pyrazole.

**Scheme 2:**
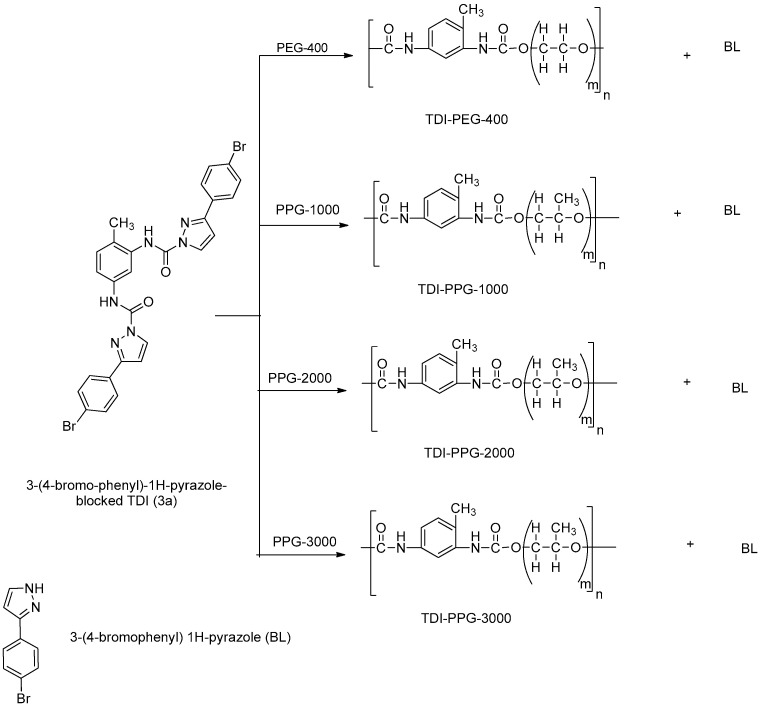
Reactions of 3-(4-bromo-phenyl)-1H-pyrazole-blocked-Toluene diisocyanate with polyols.

Toluene diisocyanate (TDI) was reacted with 3-(4-bromophenyl)-1H-pyrazole as a blocking agent to form blocked isocyanate adducts. NMR, IR, TGA and DSC were used to characterize the materials, and to investigate the deblocking temperature. At the deblocking temperature, regenerated toluene diisocyanate was reacted with polyols of different molecular weights to form polyurethane. Scheme 1 describes the blocking reactions of isocyanates with 3-(4-bromo-phenyl)-1H-pyrazole. Scheme 2 describes the reactions of blocked TDI with 3-(4-bromo-phenyl)-1H-pyrazole with polyols.

### 2.1. Synthetic Procedure of 4-(3-Bromophenyl)-1H-Pyrazole-Blocked Toluene Diisocyanate Adducts

In total, 4.48 × 10^−4^ mol (0.1 g) of the blocking agent and 2.24 mol (0.03 mL) of TDI were taken in a 50 mL single-necked round-bottomed flask along with porcelain beads and dissolved in 15 mL solvent. TDI was added with a micropipette. In total, 0.54 weight percent of Dibutyltin dilaurate was added with a micropipette using 0.001% DBTDL. A guard tube was attached to it and, after refluxing, the round-bottom flask was allowed to cool to room temperature. Then, the content of the round-bottom flask was reduced in volume and transferred to a beaker; hexane was added for precipitation and kept at room temperature, i.e., 28 °C. The precipitation was filtered, and it was washed with hexane and dried in an oven at 60 °C for 6 h. For purification, the adduct was dissolved in chloroform and precipitated with hexane at room temperature.

### 2.2. Synthetic Procedure for the Preparation of Polyurethane from 4-(3-Bromophenyl)-1H-Pyrazole-Blocked TDI Adducts

In total, 3.08 × 10^−4^ moles (0.19 g) of 3-(4-bromophenyl-1H-pyrazole-blocked-toluene-diisocyanates and 3.08 × 10^−4^ moles (0.307 g) polyethers were taken in a beaker with a narrow diameter and kept in an oven at a temperature of 240 °C for 4 h. After that, the beaker was taken out of the oven in cold conditions. Then, the sample was set in a soxhlet apparatus using 500 mL pet ether and heated for 5 h. Next, 4-(3-Bromophenyl)-1H-pyrazole-blocked TDI adduct was synthesized in different conditions such as different catalytic concentrations, solvents and reflux times, as described in Table 1; the reaction conditions of polyurethane synthesis are described in Table 2.

### 2.3. Spectroscopic and Thermal Analysis

A Nicolet Impact 400, USA spectrometer was used to record the FT-IR spectra. Before recording the compound spectra, the background spectrum was taken. ^1^H and ^13^C NMR spectra were taken with a CDCl_3_ solvent on a JEOL 500 MHz ECA instrument at room temperature. For the ^13^C NMR and DEPT-135, the spectroscopic studies were performed with an operating frequency of 106 MHz. The ppm unit is used to express the chemical shift. For the thermogravimetric analysis instrument, a Netzsch, Germany with the model name “STA 449 F3 Jupiter” was used with a heating rate of 15 °C/min, and the samples were run from 25 °C to 600 °C in an inert atmosphere. The gas flows for both the balance and samples were maintained using a nitrogen atmosphere. For the DSC analysis, a differential scanning calorimetry instrument from Netzsch with the model name “DSC 214 Polyma, Germny” was used. In the case of an organic sample, the heating rate was 10 °C/min and the temperature range was 25 °C to 300 °C. In the case of a polymer sample, the heating rate of the sample was 5 °C/min, and the temperature was from −35 °C to 280 °C. A contact angle meter version 8.0 from Holmarc, India mechatronics was used for the contact angle analysis.

## 3. Results

The reaction of TDI with 3-(4-bromophenyl)-1H-pyrazole provided the blocked adduct with a yield of 91%. Because DSC predicted the deblocking temperature at 240 °C, simultaneous polymerization was performed at this temperature. In the gelation time study, the trend was observed as TDI-PEG-400 adduct < TDI-PPG-1000~TDI-PPG-2000~TDI-PPG-3000. In case of the yield of polyurethane from 3-(4-bromophenyl)-1H-pyrazole–TDI, the trend followed as TDI-PEG-400 adduct > TDI-PPG-1000 adduct > TDI-PPG-2000 ~ TDI-PPG-3000.

### 3.1. NMR Analysis

**Figure 1:**
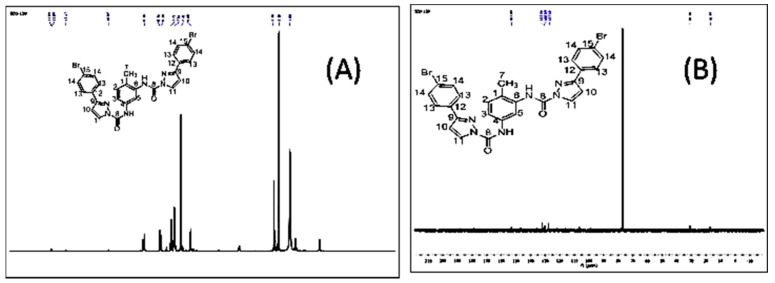
(**A**) ^1^H NMR and (**B**) ^13^C NMR of 3-(4-bromophenyl)-1H-pyrazole-blocked TDI.

**Figure 2:**
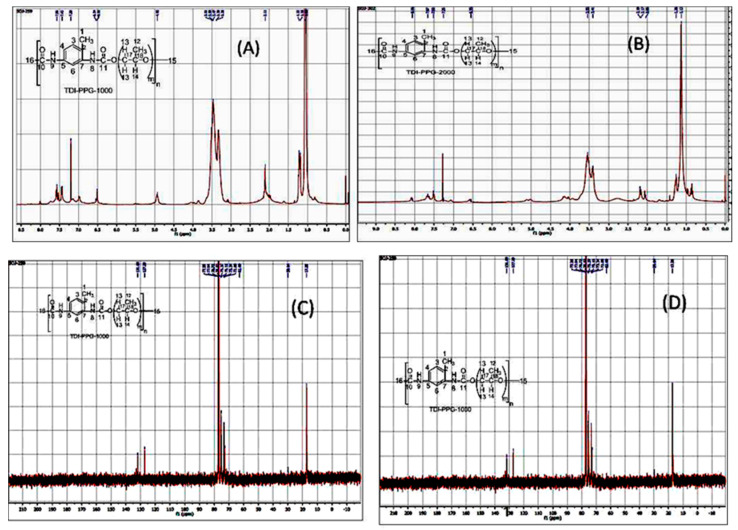
(**A**) ^1^H NMR spectrum of TDI-PPG-1000, (**B**) ^1^H NMR spectrum of TDI-PPG-2000, (**C**) ^13^C NMR spectrum of TDI-PEG-400 and (**D**) ^13^C NMR spectrum of TDI-PPG-2000.

Table 3 shows the NMR spectral data of the blocked adducts, and Figure 1 shows the ^1^H NMR and ^13^C NMR of 3-(4-bromophenyl)-1H-pyrazole-blocked TDI. Table 4 shows the NMR spectra of polyurethane from blocked TDI with 3-(4-bromophenyl)-1H-pyrazole. The NMR data supported the structures of the blocked adduct and polyurethanes (Table 3 and Table 4 and Figure 1 and Figure 2).

### 3.2. DEPT-135

**Figure 3:**
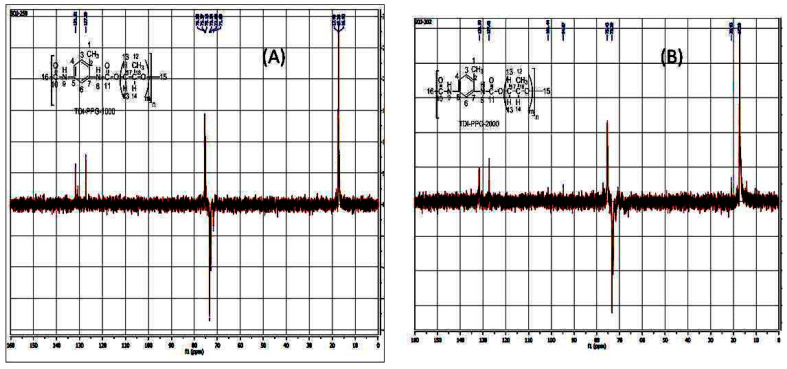
DEPT-135 spectra of (**A**) TDI-PPG-1000 and (**B**) TDI-PPG-2000.

Distortionless enhancement by polarization transfer (DEPT) has been used to separately identify the signals of methyl (CH3), methylene (CH2) and methine (CH). In DEPT spectra, methyl (CH3) and methyne (CH) carbons are up and methene (CH2) carbons are down. Table 5 represents the DEPT spectra of polyurethane from 3-(4-bromophenyl)-1H-pyrazole-blocked TDI. Figure 3 describes the DEPT-135 spectra of polyurethane.

### 3.3. FTIR Analysis

**Figure 4:**
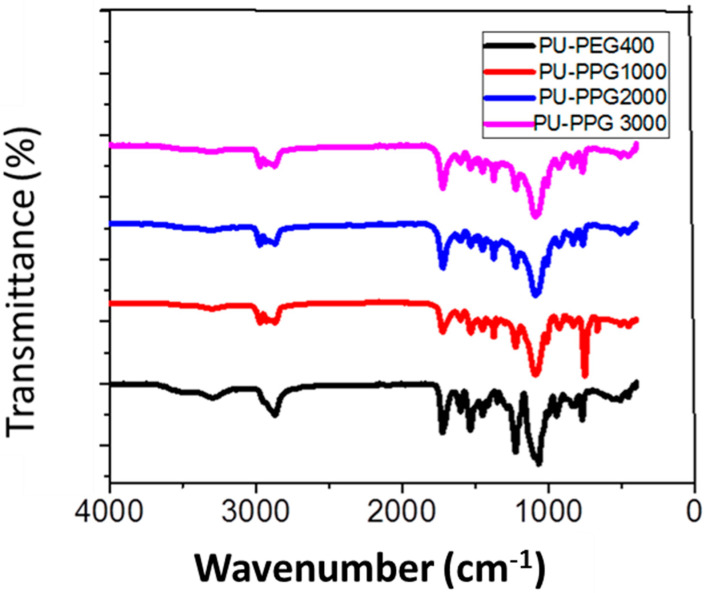
FTIR spectra of different polyurethanes formed from blocked isocyanates.

The FTIR spectra of the blocked TDI and polyurethanes are shown in Table 6. Figure 4 represents the FTIR spectra of polyurethane. The absence of a peak at 2250–2270 cm^−1^ in 3-(4-bromophenyl)-1H-pyrazole-blocked TDI implies that the isocyanate group is completely blocked by the blocking agent. The absence of a peak at 2250–2270 cm^−1^ in polyurethane implies that the isocyanate group is completely blocked in polyurethane. Figure 4 represents the FTIR spectra of TDI-PEG-400 4(a), TDI-PPG-1000 4(b), TDI-PPG-2000 4(c) and TDI-PPG-3000 4(d).

### 3.4. TGA Thermogram

**Figure 5:**
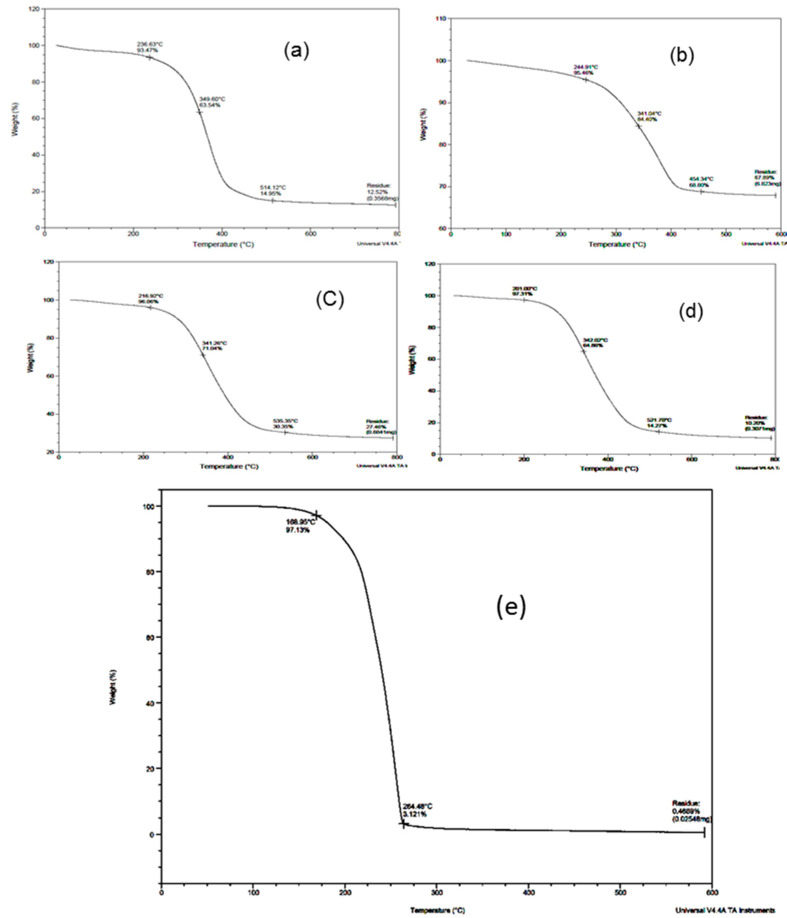
TGA thermogram of (**a**) TDI-PEG-400, (**b**) TDI-PPG-1000, (**c**) TDI-PPG-2000 and (**d**) TDI-PPG-3000; (**e**) blocked TDI.

The TGA thermograms show the starting point and ending point of the blocked isocyanates and polyurethanes that were synthesized from blocked TDI. Table 7shows the TGA thermogram data of the mentioned compounds, and Figure 5 shows the TGA thermogram of the mentioned compounds. While blocked isocyanates showed the start of their decomposition at 168 °C, all of the polyurethanes showed thermal stability above 200 °C. Furthermore, blocked isocyanates showed the endpoint of their decomposition at 264 °C, while all of the polyurethanes showed an extended range for decomposition above 400 °C. Furthermore, the majority of the compounds were decomposed above 500 °C after the formation of polyurethanes. Furthermore, while a mass loss up to 150 °C is less than 3% for blocked isocyanates, in most of the cases of polyurethanes, a mass loss of up to 3–5% was observed. This may be attributed to the water molecules physically absorbed by the polymers. The TGA thermogram shows that blocked TDI is stable up to 160 °C. Polyurethanes are stable up to between 200 °C and 240 °C.

### 3.5. DSC Thermogram

**Figure 6:**
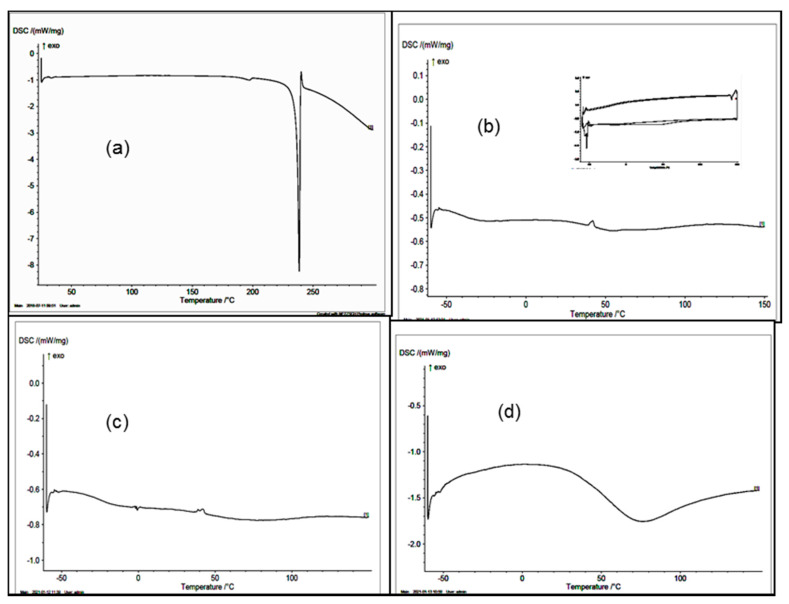
DSC thermogram of (**a**) blocked TDI, (**b**) PU-TDI-PPG-1000 (inset: two cycles), (**c**) PU-TDI-PPG-2000 and (**d**) PU-PPG-3000.

The DSC thermogram shows the deblocking behaviours of the blocked isocyanates and the thermal transitions of polyurethanes after deblocking followed by reactions with diols or polyols. As observed from Figure 6, while the deblocking temperature or melting was observed at 240 °C, the glass transitions were observed in the range of −30 to −40 °C. Interestingly, clear glass transitions were observed for polyurethanes derived from PPG 1000 and PPG 2000 in low temperatures, as was generally observed for other polyurethanes. This is usually attributed to the low crystallinity and flexibility of different chains.

### 3.6. Contact Angle Analysis

**Figure 7:**
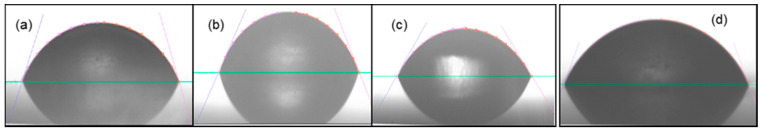
Water droplets on the coated surfaces for the determination of the contact angle of (**a**) PU-TDI-PPG-1000, (**b**) PU-TDI-PPG-2000, (**c**) PU-TDI-PPG-2000 and (**d**) blocked isocyanates.

Table 8 shows the contact angle values of the polyurethanes. Figure 7 shows water droplets on surfaces coated with different polyurethanes. As observed from the figures and tables, the contact angle data indicated the generally hydrophilic natures of the polyurethanes. Furthermore, the hydrophilicity increased with an increase of the molecular weights of the PPGs. This may be attributed to the presence of more oxygen moieties and reduced roughness [31]. It was observed by several researchers that roughness induces hydrophobicity due to the possibility of the formation of air pockets.

## 4. Discussion

It was generally observed that the gel time increased as the molecular weight of the polyol increased, and that polyurethane from polyethylene glycol showed a better yield than polyurethane from polypropylene glycol. In the case of the gelation time, PEG-polyurethane has a lower gelation time than PPG-polyurethane. The ^1^H and ^13^C NMR and DEPT-135 data supported the structures of the blocked adduct and polyurethanes. Mechanistically, the blocking of diisocyanates happens by the initial reaction of isocyanates with amine to form the intermediate compound, which can further react with polyol to form polyurethanes. For the blocked adduct, in ^1^H NMR, the peaks for the aryl rings appeared between 7–8 ppm, while the peaks for the pyrazolyl rings appeared above 8 ppm. In ^13^C NMR, the characteristic peaks for aromatic protons appeared between 100 and 120 ppm, while the characteristic peaks for carbonyl carbon appeared at 150 cm^−1^. After deblocking to produce polyurethanes, the peaks for the blocking agent disappeared expectedly, while the other characteristic aromatic protons appeared between 7 and 8 ppm. Similarly, in ^13^C NMR, the characteristic peaks for aromatic rings appeared above 100 ppm. From FTIR, the absence of a peak at 2250–2270 cm^−1^ in 3-(4-bromophenyl)-^1^H-pyrazole-blocked TDI implies that the isocyanate group is completely blocked by the blocking agent. Furthermore, the absence of a peak at 2250–2270 cm^−1^ after deblocking in the presence of PEG or PPG formation implies the successful formation of polyurethane. The TGA thermogram showed that blocked TDI was stable up to 166 °C, and that the polyurethanes were stable up to between 200 °C and 240 °C. The DSC thermogram showed the deblocking behaviours of the blocked isocyanates, indicating that at 240 °C blocked TDI deblocks. Polyurethane shows different glass transition temperatures in which the polyurethanes changes from a glassy state to rubbery state. The contact angle data showed that all of the synthesized polyurethane compounds are hydrophilic.

## 5. Conclusions

Here, 3-(4-bromo-phenyl)-^1^H-pyrazole was successfully used to block toluene diisocyanate. Spectroscopic data such as ^1^H, ^13^C NMR, DEPT-135 and FTIR confirmed the structure of the blocked TDI and the polyurethane synthesized from those blocked TDI. This type of blocking is particularly important to conveniently handle toxic substrates like TDI. Furthermore, the blocked isocyanate is stable for up to one year. The deblocking and polyurethane formation are conveniently performed in solvent-free conditions. The contact angle analysis indicated the hydrophilic nature of polyurethane.

## Figures and Tables

**Table 1:** Synthesis of 4-(3-bromophenyl)-1H-pyrazole-blocked TDI adduct.

S. No.	TDI Gram × 10^−4^	Blocking Agent (Gram)	Adduct (Gram)	Solvent	Reflux Time (Hour)	Catalyst DBTDL (wt.%)	Yield (%)
1.	3.9	0.10	0.13	CHCl_3_	3.0	0.85	91
2.	3.9	0.10	0.12	CHCl_3_	3.0	0.65	85
3.	3.9	0.10	0.12	CHCl_3_	3.0	0.50	88
4.	3.9	0.10	0.11	CHCl_3_	3.0	1.0	81

**Table 2:** Reaction conditions of polyurethane from 4-(3-bromophenyl)-1H-pyrazole-blocked TDI adduct.

S. No.	Wt. of Blocked Adduct (Gram)	Polyether	Wt of Polyether (Gram × 10^−4^)	Polyurethane	Wt of Polyurethane Gram	Yield (%)	Temp. (°C)	Gelation Time (h)
1.	0.10	PEG-400	6.44	TDI-PEG-400	0.061	95	240	3
2.	0.19	PPG-1000	3.22	TDI-PPG-1000	0.175	34	240	4
3.	0.1	PPG-2000	3.22	TDI-PPG-2000	0.13	32	240	4
4.	0.1	PPG-3000	4.83	TDI-PPG-3000	0.18	32	240	4

**Table 3:** NMR spectra of the blocked adduct.

Compound	^1^H NMR (in ppm)	^13^C NMR (in ppm)
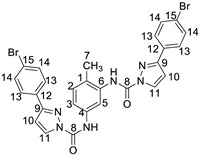	2.43 (CH_3_-7), 6.69 (2 H-10), 7.51 (H-2, H-3), 7.51 (4 H-13), 7.71(4 H-14), 8.0 (H-5), 8.21 and 8.31 (2 H-11), 9.10 (2 N-H).	16.93 (CH_3_-7), 108 (C-5), 127.18 (4 C-13), 130 (4 C-14), 152 (2 C-8).

**Table 4:** NMR spectra of polyurethane from blocked TDIwith3-(4-bromophenyl)-1H-pyrazole.

Compound	^1^H NMR (in ppm)	^13^C NMR (in ppm)
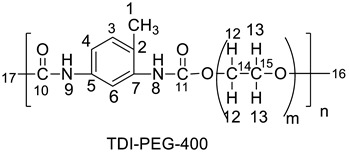	1.54, 1.19 (H-16), 2.11 (H-17), 2.24 (H-1), 3.65 (2H-13), 4.22 (2H-12), 6.97 (N-H-8), 7.42 (H-3), 7.57 (H-4), 7.69 (H-6), 8.00 (N-H-9).	17.37 (C-1), 31.25 (C-17), 61.49 (C-16), 64.32 (C-14), 67.3 (C-15), 115 (C-6), 122 (C-4), 127 (C-2), 130.7–131.7 (C-7), 136–137 (C-5), 149 (C-10), 153 (C-11).
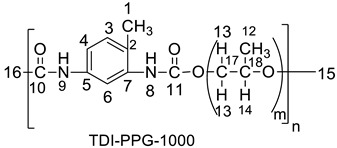	1.10 (H-15), 1.24 (H-12), 2.14 (H-1), 3.34 (H-14), 3.47 (H-13), 6.51 (H-9), 7.2 (H-3), 7.48 (H-4), 7.56 (H-6), 8.01 (H-8)	17.28 (C-15, C-1), 29.64 (C-16), 72.8 (C-15), 73 (C-17), 75 (C-18), 127 (C-4, C-6), 131.8 (C-7, C-5).
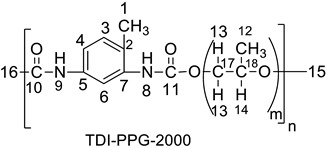	1.15 (H-15), 1.23 (H-12), 2.06, 2.26 (C-1), 3.41 (2H-13), 3.55 (H-14), 7.51 (H-3, H-4), 7.65 (H-6), 8.09 (H-8, H-9).	17.2 (C-15), 22.62 (C-1), 28.29 (C-12), 45 (C-16), 72.75 (C-17), 75 (C-18), 127 (C-4, C-6), 131 (C-7,C-5), 170 (C-10), 196 (C-11).
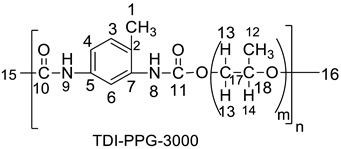	1.06 (H-16), 1.19 (H-12), 3.33 and 3.45 (2H-13), 4.12 (H-14), 7.45 (H-3, H-4), 7.54 (H-6), 8.01 (N-H-9).	17.3 (C-1), 17.4 (C-12), 22 (C-16), 31 (C-17), 73, 127.28 (C-5, C-7), 131 (C-3,C-4, C-6).

**Table 5:** DEPT-135 spectral data of polyurethane from 3-(4-bromophenyl)-1H-pyrazole-blocked TDI.

Compound	DEPT-135 (in ppm)
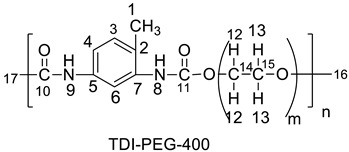	17.11 (up)[CH_3_-1], 61.28 (down)[CH_2_-14], 63.5 (down) [CH_2_-13], 77.72 (down) [CH_2_-16], 126.9 (up) [CH-3], 130.13 (up) [CH-4], 131.7 (up) [CH-6].
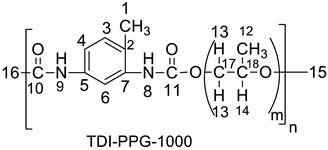	17.12 (up) [CH_3_-1], 17.4 (down) [CH_3_-12], 71.69 (down) [CH_2_-15], 72.88 (down) [CH_2_-17], 73.61 (down) [CH_2_-16], 127.29 (up) [CH-3], 130 (up) [CH-4], 131 (up) [CH-6].
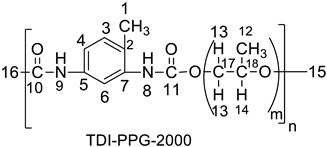	17.79 (up) [CH_3_-12], 20.9 (up) [CH_3_ -1], 73.39 (down) [CH_2_-17], 75.43 (down) [CH_2_-15], 77.30 (up) [CH-7],127 (up) [CH-3, CH-4], 131.5 (up) [CH-6].
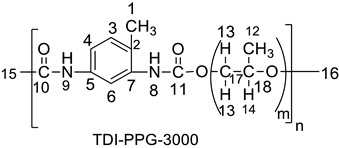	17.45 (up) [CH_3_-1, CH_3_-12], 73.33 (down [CH_2_-17], 74.95 (down) [CH_2_-16], 127.19 (up), [CH-3, CH-4], 131 (up) [CH-6].

**Table 6:** FTIR spectral data of blocked TDI and the polyurethane from it.

Compound	−NH Stretch (cm^−1^)	−NCO Stretch (cm^−1^)	−C=O Stretch (cm^−1^)	CH Stretch (cm^−1^)	C–N Stretch (cm^−1^)	C–O− Stretch (cm^−1^)	C−H Bending (cm^−1^)
Blocked TDI	3355	-	1730	3143	1230	1000	752
TDI-PEG-400	3306	-	1730	2880	1230	1081	781
TDI-PPG-1000	3306	-	1730	2868–2992	1230	1093	756
TDI-PPG-2000	3318	-	1730	2980–2868	1230	1081	769
TDI-PPG-3000	3318	-	1730	2868–2980	1230	1093	769

**Table 7:** TGA thermogram of the mentioned compounds.

Compound	Starting Point	Ending Point
Blocked TDI	168.95 °C (97.13%)	264.48 °C (3.121%)
PU-TDI-PEG-400	236.63 °C (93.47%)	514.12 °C (14.95%)
PU-TDI-PPG-1000	244.9 °C (95.46%)	454.34 °C (68.80%)
PU-TDI-PPG-2000	216.92 °C (96.06%)	535.35 °C (30.35%)
PU-TDI-PPG-3000	201.00 °C (97.31%)	521.70 °C (14.27%)

**Table 8:** Contact angle data of polyurethanes.

Compound	Contact Angle
PU-TDI-PPG-1000	66.3°
PU-TDI-PPG-2000	56.7°
PU-TDI-PPG-3000	43.0°
